# An Uncommon Presentation of Carcinosarcoma of the Stomach and a Minimally Invasive Approach for Treatment

**DOI:** 10.1155/2022/1047334

**Published:** 2022-04-07

**Authors:** Anoush Calikyan, Agedi N. Boto, Valeriia Klymenko, Imran M. Siddiqui

**Affiliations:** ^1^Frank H. Netter School of Medicine at Quinnipiac University, North Haven, CT, USA; ^2^St. Vincent's Medical Center, Department of Pathology, Bridgeport, CT, USA; ^3^St. Vincent's Medical Center, Department of Medical Oncology, Bridgeport, CT, USA; ^4^St. Vincent's Medical Center, Department of Surgical Oncology, Bridgeport, CT, USA

## Abstract

Carcinosarcoma is a rare malignant neoplasm that is composed of both epithelial and mesenchymal tumor components. Gastric carcinosarcoma is even more rare and is often diagnosed at a late stage. In this report, we investigate a case of early gastric carcinosarcoma with regional lymph node metastasis in a 78-year-old woman. The patient underwent partial gastrectomy, lymphadenectomy, and splenectomy. The tumor was confined to the gastric submucosa, and a biopsy specimen led to a histological diagnosis of carcinosarcoma with adenocarcinoma, squamous-cell carcinoma, and undifferentiated pleomorphic sarcoma components. Metastasis was present in one lymph node and displayed osteosarcomatous differentiation. Vigilant monitoring for recurrence and metastatic disease will be required for this patient.

## 1. Introduction

Carcinosarcoma is an aggressive and malignant neoplasm composed of both epithelial and mesenchymal components. By the time of diagnosis, the cancer has often metastasized to other organs due to its aggressive nature [1]. Diagnosing gastric carcinosarcoma promptly and treating the patient appropriately are critical for better outcomes and reducing recurrence.

We present the case of a 78-year-old woman with a distant history of a duodenal ulcer who was found to have 2 primary synchronous lesions of carcinosarcoma in her stomach. The case is of interest because this type of tumor rarely occurs in the gastric mucosa, particularly as a primary tumor in the stomach. Moreover, the patient was not found to have any distant metastases, highlighting the importance of early detection in the management of this high-grade sarcoma.

## 2. Case Presentation

A 78-year-old woman presented to the emergency department because of concerns about melena due to gastric bleeding, vague abdominal pain, and fatiguability. She denied hematemesis or hematochezia. She had a remote history of a duodenal ulcer, well-controlled gastroesophageal reflux disease, diverticulosis, hypertension, obesity, and hyperlipidemia. She reported moderate alcohol use.

**Figure 1:**
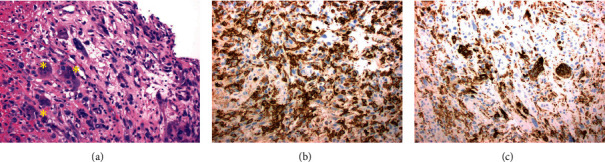
Initial endoscopic biopsy of tumor at 20x magnification. (a) Microscopic endoscopic biopsy hematoxylin and eosin stain results showed giant cells (^*∗*^) and interspersed atypical epithelioid and spindle cells. Endoscopic biopsy stained with CD45 (b) and CD68 (c) immunohistochemistry staining in lymphocytes. CD45 was positive in background lymphocytes but negative in atypical cells, and CD68 was positive in giant cells.

The physical examination yielded insignificant findings. On initial presentation, the patient was hyponatremic (131 mmol/L), anemic (hemoglobin 7.7 g/dL, hematocrit 24.5%), thrombocytic (769,000 platelets/*μ*L), leukocytic (20,100 white blood cells/*μ*L), and hypoalbuminemic (3.5 g/dL). Endoscopy showed the presence of two gastric masses, and biopsy from endoscopy revealed an undifferentiated pleomorphic sarcoma (UPS). Microscopically, the lesion was found to be an undifferentiated malignant neoplasm with epithelioid, giant cell, and focally spindled cell morphology (Figure 1(a)). Immunohistochemical staining for AE1/AE3, MOC31, CK7, CK20, p40, CDX2, CD3, CD20, PAX 5, HMB-45, S100, SMA, desmin, CD34, CD117, and dog-1 was all negative in tumor cells in the biopsied tissue. CD68 highlighted giant cells, and CD45 was negative in tumor cells (Figures 1(b) and 1(c)). Thus, the immunohistochemical profile was not consistent with a carcinoma or lymphoid malignancy. Gene assays indicated the presence of an activating PIK3CA mutation and an inactivating TP53 mutation. Morphology, immunohistochemistry, and gene assays supported a diagnosis of UPS. Endoscopic ultrasound confirmed the presence of a malignant-appearing lesion extending to the second layer (T2) with a malignant-appearing node in the splenic hilum.

Computed tomography (CT) imaging identified the presence of two intraluminal gastric masses arising from the greater curvature of the stomach that measured 3.6 and 2.2 cm in diameter. One large lymph node mass in the splenic hilum was also noted on CT scan. It was positron emission tomography (PET) avid on imaging, raising concern for metastasis to the local lymph nodes. However, on endoscopic ultrasound-guided biopsy, pathology was negative for metastatic nodal disease. Hyponatremia was thought to represent paraneoplastic syndrome of inappropriate antidiuretic hormone (SIADH).

This case was discussed in a multidisciplinary fashion by the tumor board. Owing to the patient's symptoms from anemia as well as the diagnosis of UPS, a plan was made for upfront resection and lymphadenectomy with the likelihood of en bloc splenectomy to achieve complete lymph node clearance.

The patient underwent a robotic longitudinal sleeve-like gastrectomy involving cardia, body, and antrum as well as lymphadenectomy and splenectomy. She had an uneventful postoperative course and was discharged home.

**Figure 2:**
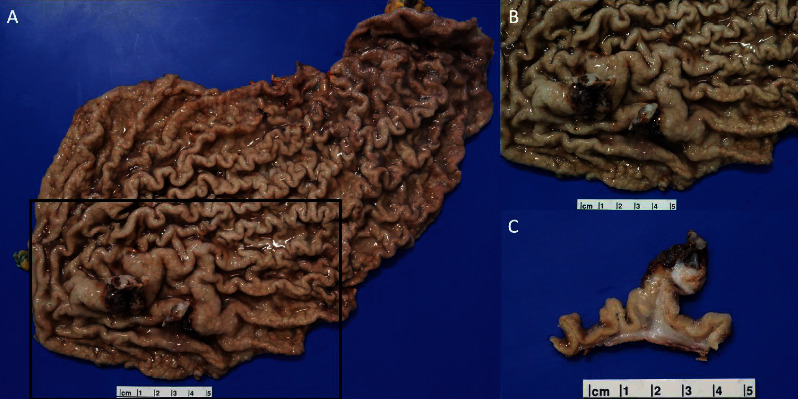
Gross lesion. (A) Image shows a fungating mass 3 cm from the margin. (B) Close-up of gross ulcerating and fungating lesion. Also seen is the diffuse granular appearance of the mucosa, which represents fundic gland polyps occurring diffusely throughout the stomach. (C) Internal view of the mass shows that the lesion was ulcerated, fungating, and growing separately from the gastric mucosa.

**Figure 3:**
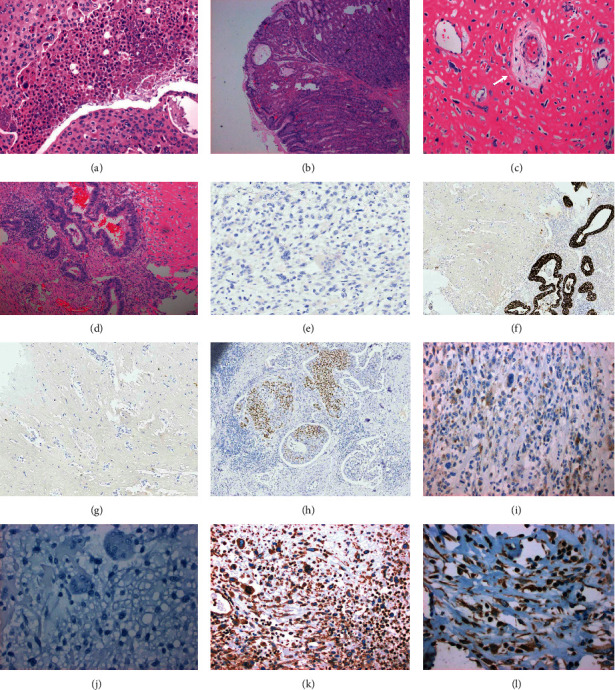
Microscopic pathology of the tumor. (a–d) Microscopic images of a fungating polyp. (a) Sheets of squamous cells displayed malignant features including large cell size and keratinization, providing evidence of squamous-cell carcinoma at 10x magnification. (b) The tumor arose in the background of a fundic gland polyp at 4x magnification. (c) Regions of the tumor and the entire lymph node metastasis displayed osteosarcomatous differentiation, evidenced by osteoid (pink) and associated atypical enlarged spindled cells at 20x magnification. (d) Evidence of both adenocarcinoma (left) and osteosarcoma (right) in the same fungating polyp tissue at 10x magnification. (e, f) AE1/AE3 staining. (e) Sarcomatous tissue of the tumor stained negative for AE1/AE3 seen at 20x magnification. (f) Adenocarcinoma component of the main tumor stained positive for AE1/AE3 imaged at 10x magnification. (g) Osteosarcoma component of the main tumor stained negative for AE1/AE3 at 10x magnification. (h) The squamous-cell carcinoma component was positive for P40 seen at 10x magnification. (i) The undifferentiated pleomorphic sarcoma was focally CD138 positive imaged at 20x magnification. (j) Beta-hCG was negative in giant cells, ruling out a choriocarcinoma component, seen at 20x magnification. (k) The undifferentiated pleomorphic sarcoma with osteoclast-like giant cells component was diffusely vimentin positive, seen at 10x magnification. (l) The osteosarcoma component of tumor was vimentin positive, imaged at 10x magnification.

**Figure 4:**
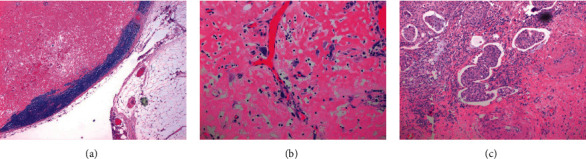
Evidence of lymph node metastasis. (a) Osteoid (pink) from osteosarcoma metastasis seen in a lymph node at low power (4x). Note the blue lymphoid cuff on the rim of the lymph node just under the capsule, identifying it as a largely replaced lymph node. (b) Osteoid and atypical spindled cells from osteosarcoma were found in a distant lymph node (higher power of (a) at 20x magnification). (c) Squamous lymphovascular invasion in the main tumor at 10x magnification. Squamous-cell tumor plugs displayed invasion in the lymphatic system.

The tumor was located in the body of the stomach and measured 9 cm. Grossly, gastric lesions were found to be ulcerating and fungating, and the gastric mucosa was diffusely covered in fundic gland polyps (Figures 2(a)–2(c)). The gastric lesion was limited to the submucosa. Eleven lymph nodes were dissected, one of which was found to be positive for metastasis and measured 2.0 cm × 1.9 cm × 1.4 cm. This case was thus classified as American Joint Committee on Cancer (AJCC) stage IB. Pathology revealed pT1bN1 carcinosarcoma of the stomach. The biopsy showed not only evidence of UPS with osteoclast-like giant cells and osteosarcoma but also multiple lineages of differentiation including adenocarcinoma and squamous-cell carcinoma (Figures 3(a)–3(d)), which was consistent with a diagnosis of carcinosarcoma. The immunoprofile of the resected tumor showed that the squamous-cell and adenocarcinoma components were both positive for AE1/AE3 and CK7 (Figures 3(e)–3(g)). CDX2 was focally positive in the adenocarcinoma component and predominantly negative in the squamous-cell and sarcomatous components. CK20 was focally positive in the adenocarcinoma component and predominantly negative in the squamous-cell carcinoma component. P40 was positive in the squamous-cell carcinoma component but negative in the adenocarcinoma and sarcomatous components (Figure 3(h)). SATB2 was negative in the sarcomatous component. HMB45, c-kit, and dog-1 were negative. CD138 was focally positive in the UPS, adenocarcinoma, and squamous-cell carcinoma components (Figure 3(i)). Beta-human chorionic gonadotropin (*β*-hCG) was negative in giant cells, ruling out a choriocarcinoma component (Figure 3(j)). The sarcomatous component (both pleomorphic undifferentiated sarcoma with osteoclast-like giant cells and osteosarcoma components) was positive for vimentin (Figures 3(k) and 3(l)) and negative for AE1/AE3 (Figures 3(e) and 3(g)). Thus, the immunohistochemical profile on the resection supported a diagnosis of carcinosarcoma. Microscopically, lymph node metastasis displayed osteosarcomatous differentiation (Figures 4(a) and 4(b)). Pathology also revealed evidence of lymphovascular invasion (Figure 4(c)).

The patient underwent adjuvant chemotherapy (platinum based) after discussion by the tumor board. The case was discussed with national experts who agreed with the plan of care, given the rarity of the disease.

## 3. Discussion

Carcinosarcoma is a rare, malignant, and biphasic tumor that has aspects of both carcinoma (a cancer of epithelial tissue) and sarcoma (a cancer of connective tissue). It is often of müllerian origin and arises in the uterus, ovary, and fallopian tube, but it has also been reported to occur in the esophagus, thyroid, lungs, larynx, breasts, and urinary tract [1, 2]. While already rare, carcinosarcoma of the gastric mucosa is much rarer, with a literature review citing fewer than 100 reported cases of gastric carcinosarcoma [3].

The pathogenesis of gastric carcinosarcoma is still debated, but there are 2 main theories. The first includes the bi-clonal origin hypothesis, which suggests that 2 separate tumor cell clones collide. The second theory is the monoclonal origin hypothesis, which indicates that a stem cell is able to differentiate into both epithelial and mesenchymal cells [4]. Sarcomas rarely metastasize to lymph nodes, but they are more likely to do so if they have an epithelioid subtype [5]. In this case, osteosarcoma was found to have metastasized to a lymph node, thus supporting the monoclonal origin theory.

In most patients, the carcinomatous portion of the carcinosarcoma involves adenocarcinoma, but a few reports have described an adenocarcinoma with neuroendocrine differentiation [6] or adenosquamous carcinoma [7], as in our case. The sarcomatous portion of carcinosarcomas is variable, but they can include leiomyosarcoma, rhabdomyosarcoma, and osteosarcoma or can be undifferentiated [8]. The sarcomatous components in the current case included osteosarcoma and UPS with osteoclast-like giant cells. The presence of bone differentiation caused gastric adenocarcinoma with osseous differentiation to be included in the differential diagnosis; however, in that entity, the osseous component is benign, while the osseous component in our case had malignant spindled cells [9]. The presence of osteoclast-like giant cells associated with a carcinoma raised the possibility of primary gastric choriocarcinoma in the differential diagnosis; however, that was not consistent with the observed hCG negativity and cytokeratin negativity in the sarcomatous component, as seen in Figures 3(e)–3(j) [10]. The presence of discohesive individual cells with rare CD138 positivity (Figure 3(i)) raised the possibility of a gastric carcinoma with plasmacytoid morphology [11]; however, the discohesive cells staining weakly for CD138 were cytokeratin negative (Figure 3(e)) and vimentin positive (Figure 3(k)), supporting the diagnosis of carcinosarcoma.

Clinical features of this neoplasm are nonspecific but most commonly include epigastric pain, weight loss, and melena [3]. The treatment for most patients involves surgical resection by total gastrectomy [1], while some patients undergo chemotherapy [4]. Our case was unique in that the patient was treated with a robotic partial gastrectomy, rather than a total gastrectomy. This approach was used to achieve improved functional outcomes in this elderly patient, while also preserving oncologic principles of wide margins and lymphadenectomy. The unique pathology also made this case very interesting and challenging to treat. Close surveillance will be needed postoperatively to monitor for metastases or recurrence of the gastric tumor. A review of the literature did not reveal a standard postoperative treatment of this disease.

The prognosis for patients with carcinosarcoma remains quite poor, with a mean survival period estimated to be 7–10 months, with 50% of recurrences occurring within the first year postoperatively [12]. To our knowledge, only 6 cases [1, 4, 13–15], including ours, have been diagnosed at an early stage thus far. Interestingly, 4 of the 6 cases showed regional lymph node metastasis, demonstrating the aggressive nature of this tumor. Early identification remains crucial to preventing recurrences and metastasis of this aggressive tumor.

## 4. Conclusion

Gastric carcinosarcoma is rare and has a poor prognosis. Although surgical resection may improve outcomes, the tumor is aggressive and has often already metastasized before diagnosis. The present study explored the clinical findings, diagnosis, and prognosis of gastric carcinosarcoma. Continued research in this area is required to lessen the burden of this rare disease.

## Data Availability

The endoscopy and biopsy data used to support the findings of this study are included within the article.
